# Does partial replacement of corn with glycerin in beef cattle diets affect in vitro ruminal fermentation, gas production kinetic, and enteric greenhouse gas emissions?

**DOI:** 10.1371/journal.pone.0199577

**Published:** 2018-06-21

**Authors:** Pedro Del Bianco Benedeti, Mozart Alves Fonseca, Teshome Shenkoru, Marcos Inácio Marcondes, Eduardo Marostegan de Paula, Lorrayny Galoro da Silva, Antonio Pinheiro Faciola

**Affiliations:** 1 Department of Animal Sciences, Universidade Federal de Viçosa, Viçosa, Minas Gerais, Brazil; 2 Department of Agriculture, Nutrition, and Veterinary Sciences, University of Nevada, Reno, Nevada, United States of America; 3 Department of Animal Sciences, Universidade do Estado de Santa Catarina, Chapecó, Santa Catarina, Brazil; 4 Department of Animal Sciences, University of Florida, Gainesville, Florida, United States of America; University of Illinois, UNITED STATES

## Abstract

Five in vitro experiments were conducted with the following objectives: 1) To evaluate the ruminal fermentation of three different single ingredients: corn, glycerin, and starch (**Exp. 1** and **2)**; 2) To assess effects of partially replacing corn with glycerin in beef cattle diets on ruminal fermentation pattern (**Exp. 3** and **4**); and 3) To evaluate the effects of glycerin inclusion on the extension of ruminal DM digestibility of feeds with high (orchard hay) and low (corn) fiber content (**Exp. 5**). For **Exp. 1** and **2**, two in vitro systems (24-bottle Ankom^RF^ and 20-serum bottles) were used in four consecutive fermentation batches to evaluate gas production (GP), fermentation profiles, enteric methane (CH_4_), and carbon dioxide (CO_2_) of corn, glycerin, and starch. The 24 h total GP, acetate concentration, and acetate: propionate ratio decreased only when glycerin was added to the diet (*P* < 0.01). The 48-h total GP and metabolizable energy were greatest for corn (*P* < 0.01), and similar between glycerin and starch. The starch treatment had the lowest total volatile fatty acids concentration (*P* = 0.01). Glycerin had greatest CH_4_ production, lag time, and maximum gas volume of the first pool (*P* < 0.05). However, the maximum gas volume of the second pool was greatest for corn (*P* < 0.05), and similar between glycerin and starch. The starch treatment had the greatest specific rates of digestion for first and second pools (*P* < 0.05). Production of CO_2_ (mL/g) was greater for corn (*P* < 0.01), but similar for glycerin and starch. For **Exp. 3** and **4**, the same systems were used to evaluate four different levels of glycerin [0, 100, 200, and 300 g/kg of dry matter (DM)] replacing corn in beef cattle finishing diets. Glycerin levels did not affect 24 and 48 h total GP, CH_4_, and CO_2_ (*P* > 0.05). The inclusion of glycerin linearly decreased acetate concentration (*P* = 0.03) and acetate: propionate ratio (*P* = 0.04). For **Exp. 5**, two Daisy^II^ incubators were used to evaluate the in vitro dry matter digestibility (IVDMD) of the following treatments: orchard hay; corn; orchard hay + glycerin; and corn + glycerin. Glycerin inclusion decreased orchard hay IVDMD (*P* < 0.01) but did not affect corn IVDMD (*P* > 0.05). We concluded that, under these experimental conditions, glycerin has similar energy efficiency when used in replacement of corn and included at up to 300 g/kg in beef cattle diets.

## Introduction

The expansion of the biodiesel industry has increased glycerin supply [1] making it a feasible alternative to corn for feeding cattle, even when glycerin price was up to 10% greater than corn price [2]. Glycerin is an organic compound belonging to the alcohol group, that can increase the glucogenic potential of beef cattle finishing diets [3]. Glucose is an important carbon source used for fatty acid synthesis [4], which is especially significant for marbling in finishing animals. Therefore, estimating glycerin metabolizable energy (ME), as well as comparing it to other well-known energy sources such as corn and starch is important to assist nutritionists to effectively include it in beef cattle finishing diets.

Previous research showed that glycerin may reduce methane (CH_4_) production [5], possibly due to a shift towards propionate production, because pathways toward propionate formation act as a hydrogen sink [6, 7] reducing the availability of hydrogens for CH_4_ formation. Therefore, glycerin has the potential to be used as a sustainable alternative to replace corn as energy source in beef cattle finishing diets. Previous in vitro [3, 8] and in vivo [9, 10] studies have reported positive effects on ruminal fermentation when glycerin was included in beef cattle finishing diets. However, there is still controversy regarding the effects of glycerin on total gas production (GP) and enteric CH_4_ production [11], NDF digestibility [2], the recommended levels of glycerin inclusion in beef cattle finishing diets [9, 10], and its impact on ruminal fermentation of high fiber feeds [12, 13].

We hypothesized that glycerin fermentation would be similar to starch and could partially replace corn as dietary energy source in beef cattle finishing diets at up to 300 g/kg (DM basis), without compromising ruminal fermentation, total VFA concentration and profile, total GP, enteric CH_4_ and carbon dioxide (CO_2_) production, and in vitro dry matter digestibility (IVDMD). Therefore, the objectives of this research were: 1) To evaluate the ruminal fermentation of three different single ingredients: corn, glycerin, and starch (**Exp. 1** and **2**); 2) To assess effects of partially replacing corn with glycerin in beef cattle diets on ruminal fermentation pattern (**Exp. 3** and **4**); and 3) To evaluate the effects of glycerin inclusion on the extension of ruminal DM digestibility of feeds with high (orchard hay) and low (corn) fiber content (**Exp. 5)**.

## Materials and methods

### Location and ethical approval

All experiments were conducted at the University of Nevada, Reno. This study was carried out in strict accordance with the recommendations of the Institutional Animal Care and Use Committee (IACUC) of the University of Nevada, Reno. Animal care and handling protocol were approved by the IACUC (Protocol Number: 00588). Surgically prepared animals were the same used in previous studies [3] in which surgery was performed under anesthesia (lidocaine hydrochloride and butorphanol tartrate), and all efforts were made to minimize suffering, as described in Del Bianco Benedeti et al. [3].

### Ingredients evaluation: Corn, glycerin, and starch (Exp. 1 and 2)

First, two experiments were performed for individual feed evaluation. Treatments were three different single ingredients: corn, glycerin [Purity of 997 g/kg of organic matter (OM); Nature’s Oil, Streetsboro, OH, USA], and starch. Ingredients were incubated in four 48-h fermentation incubations to assess the in vitro GP profiles (**Exp.1**) and enteric CH_4_ and CO_2_ production (**Exp. 2**). For **Exp.1**, each fermentation batch contained 7 laboratory replicates of each ingredient and 3 blanks (rumen/buffer solution only), totaling 96 observations. For **Exp.2**, there were 5 laboratory replicates of each ingredient and 5 blanks per fermentation batch, totaling 80 observations.

### Rumen fluid collection and rumen/buffer solutions preparation

For **Exp. 1** and **2**, rumen fluid was collected from two Aberdeen Angus steers, cannulated in the rumen (Average body weight of 500 kg). Steers were maintained on a total mix diet composed of 400 g/kg orchard grass hay, 470 g/kg dry ground corn, 100 g/kg soybean meal, and 30 g/kg mineralized salt [dry matter (DM) basis]. Two h after feeding, 2000 mL of rumen fluid were collected, immediately filtered through 4 layers of cheesecloth and kept into pre-warmed thermal containers and transported to the lab.

The buffer mineral solutions of both experiments were prepared according to Menke and Steingass [14], with the addition of sodium sulfite and L-cysteine [15]. The buffer solution was kept in a water bath at 39^°^C and purged continuously with nitrogen (N_2_) for 30 min. Resazurin was used as color indicator to control the buffer pH and N_2_ saturation (oxidation-reduction potential). The rumen fluid was mixed with the buffer solution (1:2 v/v) in water bath at 39^°^C under anaerobic conditions by flushing N_2_ [14].

### In vitro gas production

A 24-bottle system (Ankom^RF^ GP System, Ankom Technology, NY, USA) equipped with pressure sensors wireless connected to a computer, was used (**Exp. 1**). Each bottle (620 mL) was filled with 0.5 g of each ingredient. Samples were hydrated with deionized water to avoid particle dispersion. Bottles were inoculated with 75 mL of rumen/buffer solution keeping the headspace of bottle continuously flushed with N_2_. After inoculation, bottles were closed and placed in the air-ventilated shaker incubator (Innova 4400 incubator shaker; New Brunswick Scientific, Edison, NJ, USA) under controlled temperature (39^°^C) and agitation (83 rpm). The data acquisition software (Gas Pressure Monitor, Ankom technology, NY, USA) was set to monitor the cumulative pressure every 5 minutes and data was recorded every 60 minutes for 48 h. Valves were set to automatic release the gas when the pressures reached 3.4 kPa [16].

The cumulated gas pressures at 24 and 48 h were converted into mL according to Tagliapietra et al. [16] as GP, mL = (P_c_/P_o_) x V_o_, where P_c_ is the cumulated pressure change (kPa) in the bottle headspace; V_o_ is the bottle headspace volume (545 mL), P_o_ is the atmospheric pressure read by the equipment at the beginning of the measurement. The bottles’ final GP volumes were corrected for inoculum contribution by subtracting the final GP of the blank bottles. For total GP over time, the cumulative pressure values were adjusted to assess biological values using the following dual-pool model [17]: V_t_ = (V_1_ / (1 + (EXP(2 + 4 x (C_1_ x (L–Time)))))) + (V_2_ / (1 + (EXP(2 + 4 x (C_2_ x (L–Time)))))), were: V_t_ = gas volume produced up to the specific time, mL; V_1_ and V_2_ = maximum gas volume achieved from complete digestion of each pool, mL; C_1_ and C_2_ = specific rate of digestion of each pool, h^-1^; Lt = lag time, h. The ME was calculated according to Menke and Steingass [14], with lipid content ignored [18], as ME (MJ/kg DM) = 2.20 + (0.1357 × GP_200_) + (0.0057 × CP) where GP_200_ (mL/200 mg of DM incubated) is the GP measured at 48h. The solution pH was measured (Accumet™ AP61, Fisher Scientific, Atlanta, GA) at the beginning and at the end of each incubation (48 h).

### Enteric CH_4_ and CO_2_

As the Ankom^RF^ is a vented system, a second closed system composed by serum bottles was used to investigate both in vitro enteric CH_4_ and CO_2_ production (**Exp. 2**). Each serum bottle (155 mL) was filled with 0.2 g of each ingredient. Bottles were inoculated with 20 mL of rumen/buffer solution keeping the headspace of bottle continuously flushing with N_2_. After inoculation, bottles were sealed with butyl rubber stoppers and aluminum caps, and then placed into an air-ventilated shaker incubator (39^°^C). At the end of each fermentation batch, CO_2_ and CH_4_ production were measured from the headspace using a Gow Mac thermal conductivity series 580 gas chromatograph (Gow Mac Instrument, Bridgewater, NJ) equipped with a Porapak Q (Supelco) column (60°C, 30 mL/min of helium (999.9 mL/L) as the carrier gas). The bottles’ enteric CH_4_ and CO_2_ productions were corrected for inoculum contribution by subtracting the final GP of the blank bottles. The solution pH was measured (Accumet™ AP61, Fisher Scientific, Atlanta, GA) at the beginning and at the end of each incubation (48 h).

### Volatile fatty acids (VFA) and ammonia-nitrogen (NH_3_-N)

For both experiments, subsamples of 10 mL from the rumen/buffer solution before incubation and from each bottle at 48 h were filtered through four layers of cheesecloth. Then, 0.2 mL of a 500 mL/L H_2_SO_4_ solution was added for determination of NH_3_-N and VFA. The VFA concentrations were determined using gas chromatography (Varian Model 3800; Varian, Inc., Walnut Creek, CA; equipped with a glass column [180 cm x 4 mm i.d.]) packed with GP 10% SP-1200/1% H_3_PO_4_ on 80/100 Chromosorb WAW (Supelco, Bellefonte, PA), and N_2_ was used as a carrier gas at a flow rate of 85 mL/min^-1^ [19]. The NH_3_-N concentration was determined by colorimetry as described by Chaney and Marbach [20]. The total VFA and NH_3_-N concentrations were calculated subtracting the values measured on the initial content of the components in the rumen/buffer solution from the final concentrations of each bottle [21].

### Diets evaluation: Different glycerin levels in beef cattle finishing diets (Exp. 3 and 4)

After ingredients evaluation, two other experiments were conducted to assess the effects of replacing dry ground corn with glycerin in diets. Then, there were four treatments: inclusion of 0, 100, 200, and 300 g/kg (DM basis) of glycerin replacing corn in beef cattle finishing diets. Treatments were incubated in four 48-h fermentation incubations to assess the in vitro GP profiles (**Exp. 3**) and enteric CH_4_ and CO_2_ production (**Exp. 4**). The diets were composed of 200 g/kg orchard hay and 800 g/kg concentrate (DM basis). For **Exp.3**, each fermentation batch contained 5 laboratory replicates of each diet and 4 blanks, totaling 96 observations. For **Exp.4**, there were 5 laboratory replicates of each diet and 5 blanks per fermentation batch, totaling 100 observations. The **Exp. 3** was performed using similar methods (system, procedures, and evaluated variables) to that used for **Exp. 1,** except treatments. Similarly, **Exp. 4** followed **Exp. 2** methods, all but treatments.

### In vitro dry matter digestibility (Exp. 5)

The **Exp. 5** rumen fluid collection procedures were similar to the previous four experiments. However, approximately 300 g of rumen solid particles were also added to the containers. For the inoculum preparation, the rumen content was blended for 2 min, followed by filtering through 4 layers of cheesecloth [22]. The buffer mineral solution was prepared following the equipment manual and the pH was adjusted to 6.8 when needed [22]. After preparation, 1600 mL of buffer solution was added in each vessel, which were placed into Daisy^II^ incubator and kept at 39^°^C for 30 min. Then, 400 mL of rumen inoculum was added in each vessel under anaerobic conditions.

Two systems of four 4-L digestion vessels (Daisy^II^ system, Ankom technology, NY, USA), equipped with slow rotation and temperature controller were used in four consecutive 48-h fermentation batches. The incubations consisted of 8 jars distributed into two fermenters, which were run simultaneously in a replicated 4 x 4 Latin square arrangement. Each of the four treatments were applied to one of the jars, totaling 8 replicates per treatment, which were: orchard hay, corn, orchard hay + glycerin, and corn + glycerin.

Corn and orchard hay were individually weighed (0.4 g/bag) into filter bags (F57, Ankom technology, Macedon, NY, USA), which were heat-sealed and placed into the digestion vessels. Each vessel received 6 bags of one of the treatments plus 2 bags with no samples (blanks) and 2000 mL of rumen/buffer solution. Then, jars with glycerin treatments were inoculated with 1.0 g of glycerin. After inoculation, vessels were closed and then placed into the incubator with temperature at 39.5^°^C for 48 h. At the end of incubation, bags were rinsed with cold water and analyzed for DM [22]. The IVDMD was calculated as IVDMD, g/kg DM = (100 –[W_3_ - (W_1_ x C_1_)] x 100 / (W_2_ x DM / 10)) x 10, where: W_1_ = bag tare weight, W_2_ = sample weight, W_3_ = final bag weight after in vitro incubation, C_1_ = blank bag correction (final oven-dried weight/original blank bag weight), DM = g/kg dry matter.

### Chemical analyses

All the ingredients used in this study were ground through a 2-mm screen (Wiley mill; Thomson Scientific Inc., Philadelphia, PA) for all incubations and analysis performed. Samples were analyzed for DM (method 934.01), ash (method 938.08), crude protein (CP; Leco CN-628 Series Determinator; method 990.13), and ether extract (EE; method 920.85) according to AOAC [23]. The OM was calculated as the difference between DM and ash contents. For neutral detergent fiber assayed with a heat stable amylase and expressed exclusive of residual ash (aNDFom), samples were treated with alpha thermo-stable amylase omitting sodium sulfite according to Van Soest et al. [24], and adapted for the Ankom200 Fiber Analyzer (Ankom Technology, Macedon, NY). Ingredient proportion and chemical composition of the experimental diets and feeds are presented in Table 1.

**Table 1:** Ingredients and chemical composition of experimental diets and feeds.

Item^1^	Glycerin, g/kg						Corn	Glycerin	Starch	Orchard hay
	0		100	200		300				
Ingredient, g/kg DM										
Orchard hay	200		200	200		200	-	-	-	-
Dry ground corn	724		604	484		364	-	-	-	-
Glycerin^2^	0.00		100	200		300	-	-	-	-
Soybean meal	76.3		96.3	116		136	-	-	-	-
Composition									
DM, g/kg	894		900	906		912	879	947	952	942
OM, g/kg DM	974		974	974		974	990	1000	1000	929
aNDFom, g/kg DM	205		191	177		163	134	-	-	509
CP, g/kg DM	135		135	135		135	93.8	-	-	121
EE, g/kg DM	28.1		24.3	20.4		16.5	33.3	-	-	17.3

^1^aNDFom = neutral detergent fiber assayed with a heat stable amylase and expressed exclusive of residual ash; CP = crude protein; DM = dry matter; EE = ether extract; OM = organic matter.

^2^Purity of 997 g/kg (OM basis; Nature’s Oil, Streetsboro, OH, USA).

### Statistical analysis

All results were tested for normality [25] and followed a normal distribution (*P* > 0.05). For **Exp. 1** through **Exp. 4,** data were collected and analyzed according to a randomized complete block design using mixed models methodology with ingredients considered fixed factors (2, 2, 3, and 3 degrees of freedom for **Exp. 1**, **Exp. 2**, **Exp. 3**, and **Exp. 4**, respectively), and fermentation batch as random factor (3 degrees of freedom for **Exp. 1** through **Exp. 4**). For **Exp. 5**, data were collected and analyzed following a replicated 4 x 4 Latin square design. All statistical procedures were carried out using SAS 9.4 for Windows (Statistical Analysis System Institute, Inc., Cary, NC, USA) with significance declared at *P* ≤ 0.05, through MIXED and GLIMMIX procedures. For **Exp. 1** through **Exp. 4,** experimental units were considered to be the mean of the bottles within ingredients/diets in each fermentation batch (true replicate), whereas for **Exp. 5,** experimental units were considered to be the jars within incubators in each fermentation batch (true replicate). For total GP over time data, logistic nonlinear functions for two pools and a discrete lag [17] were adjusted for ingredients (**Exp. 1**; corn, glycerin, and starch) as well as diets (**Exp. 3**; glycerin levels), in order to compare possible differences in fermentation profiles. Parameters of the nonlinear functions were then compared by means of sum of squares reduction test and differences were declared at *P* ≤ 0.05.

## Results

### Ingredients evaluation: Corn, glycerin, and starch (Exp. 1 and 2)

**Fig 1:**
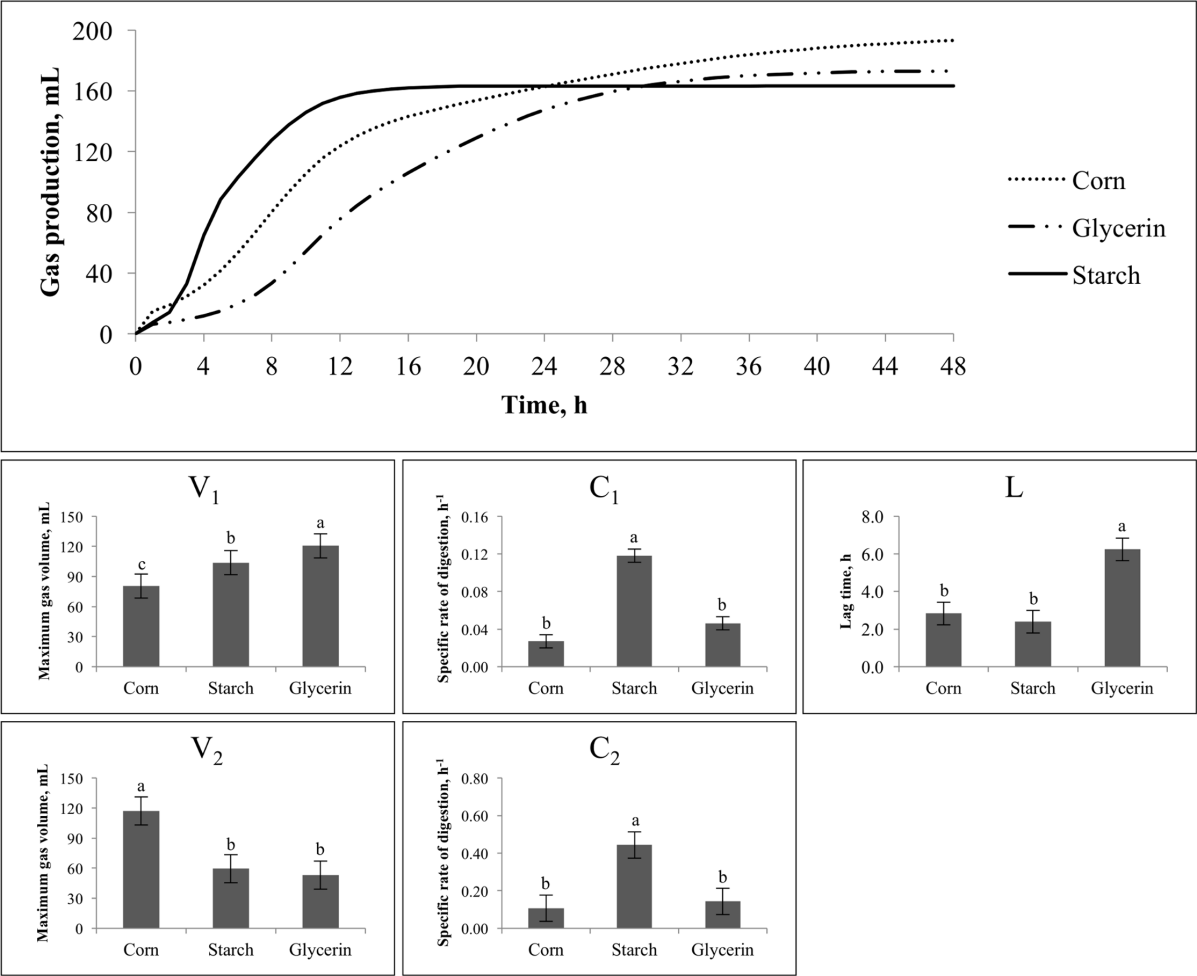
Effects of different ingredients on in vitro gas production and kinetic variables^1^ of gas production in Exp. 1. ^a,b,c^Means with different superscripts are different (P < 0.05). ^1^V1 and V2 = Maximum gas volume of each pool, mL; C1 and C2 = Specific rate of digestion of each pool, h-1; Lt = lag time, h.

For **Exp. 1**, the first 24 h of total GP (mL/g DM) indicated that corn and starch did not differ, but glycerin had the lowest values (*P* < 0.01; Table 2). However, at the end of the 48-period, starch and glycerin reached similar overall GP whereas corn was the greatest (*P* < 0.01). The lag time was longest for glycerin (*P* < 0.05), while corn and starch did not differ (Fig 1). Also, glycerin had greatest GP on the first pool (V_1_; *P* < 0.05), but smallest second pool (V_2_; *P* < 0.05). Starch, corn, and glycerin reached the maximum gas volume of the first pool at 600h, 800h, and 1830h after the beginning of the fermentation, respectively. Starch had intermediary values for both pools whereas corn had a smaller V_1_ but a greater V_2_ (*P* < 0.05). Specific rates of digestion for first and second pools (C_1_ and C_2_) were faster for starch (*P* < 0.05) but similar for corn and glycerin. For **Exp. 2**, glycerin had the greatest CH_4_ (mL/L and mL/g) production (*P* < 0.01), but the lowest CO_2_ (mL/L) production (*P* < 0.01; Table 2). When expressed in mL/g DM, CO_2_ was greatest for corn, and similar for glycerin and starch. The metabolizable energy was greatest for corn (*P* < 0.01), but similar for glycerin and starch.

**Table 2:** Effects of different ingredients on total gas production (GP), metabolizable energy (ME), and enteric methane (CH_4_) and carbon dioxide (CO_2_) production in GP systems.

Item^1^	Treatments			SEM	*P*-value
	Corn	Starch	Glycerin		
**Exp. 1**					
24h total GP, mL/g DM	324^a^	324^a^	287^b^	19.1	< 0.01
48h total GP, mL/g DM	384^a^	344^b^	354^b^	24.1	< 0.01
ME^2^, MJ/kg DM	12.7^a^	11.5^b^	11.8^b^	0.65	< 0.01
**Exp. 2**					
CH_4_, mL/L	52.2^b^	51.6^b^	89.2^a^	4.20	< 0.01
CH_4_, mL/g DM	9.71^b^	8.49^b^	13.6^a^	1.67	< 0.01
CO_2_, mL/L	309^a^	301^a^	265^b^	12.9	< 0.01
CO_2_, mL/g DM	57.1^a^	48.5^b^	40.8^b^	6.85	< 0.01

^a,b^Means with different superscripts in the same row are different (P < 0.05).

^1^DM, dry matter; SEM, standard error of the mean.

^2^ME (MJ/kg DM) = 2.20 + (0.1357 × GP_200_) + (0.0057 × CP) where GP_200_ (mL/200 mg of DM incubated).

The effects of ingredients on ruminal variables are presented in Table 3. For Exp. 1, starch had lower pH and NH_3_-N compared to glycerin and corn (*P* < 0.01). Starch had lower total VFA concentration compared to glycerin (*P* = 0.01) although did not differ from corn. Acetate was lowest for glycerin (*P* < 0.01) whereas propionate was lowest for corn (*P* < 0.01). Glycerin had the greatest butyrate and valerate concentrations (*P* < 0.01) although had the lowest (*P* < 0.01) acetate: propionate ratio, *iso*-butyrate as well as *iso*-valerate molar proportions; followed by starch and corn, respectively. For **Exp. 2**, starch also had lower pH than glycerin and corn (*P* < 0.01). Tested ingredients had no effect on total VFA (*P* = 0.50), propionate (*P* = 0.86), *iso*-butyrate (*P* = 0.10), and *iso*-valerate (*P* = 0.08) concentrations. Acetate concentration (*P* < 0.01) and acetate: propionate ratio (*P* = 0.01) were greater for corn and starch, whereas butyrate and valerate concentrations were greater for glycerin (*P* = 0.01).

**Table 3:** Effects of different ingredients on ruminal variables in gas production systems.

Item^1^	Treatments				SEM		*P*-value
	Corn	Starch	Glycerin				
**Exp. 1**							
Final pH	5.89^a^	5.67^b^	5.93^a^		0.13		< 0.01
Total VFA, mM/g DM	15.3^ab^	14.4^b^	17.4^a^		3.67		0.01
VFA profile, mol/100 mol							
Acetate	35.8^a^	35.2^a^	24.8^b^		0.42		< 0.01
Propionate	24.8^b^	29.5^a^	30.0^a^		1.43		< 0.01
Butyrate	23.0^b^	21.4^b^	28.9^a^		1.27		< 0.01
Valerate	6.26^b^	5.67^c^	8.34^a^		0.41		< 0.01
*Iso-*butyrate	3.13^a^	2.62^b^	2.40^c^		0.09		< 0.01
*Iso-*valerate	7.09^a^	5.61^b^	5.63^b^		0.18		< 0.01
Acetate: propionate	1.46^a^	1.23^b^	0.86^c^		0.07		< 0.01
NH_3_-N, mg/100 mL	18.9^a^	10.5^b^	16.6^a^		2.11		< 0.01
**Exp. 2**							
Final pH	5.22^a^	5.00^b^	5.37^a^		0.07		< 0.01
Total VFA, mM/g DM	19.8	16.7	19.3		3.16		0.50
VFA profile, mol/100 mol							
Acetate	30.7^a^	35.1^a^	18.3^b^		1.29		< 0.01
Propionate	27.5	29.2	28.3		2.37		0.86
Butyrate	29.1^b^	25.4^b^	39.6^a^		1.96		0.01
Valerate	5.32^b^	4.46^b^	7.94^a^		0.62		0.01
*Iso-*butyrate	2.26	1.92	1.80		0.16		0.10
*Iso-*valerate	5.15	4.00	4.09		0.44		0.08
Acetate: propionate	1.17^a^	1.23^a^	0.65^b^		0.12		0.01

^a,b,c^Means with different superscripts in the same row are different (*P* < 0.05).

^1^DM, dry matter; NH_3_-N, ammonia nitrogen; SEM, standard error of the mean; VFA, volatile fatty acids.

### Diets evaluation: Different glycerin levels in beef cattle finishing diets (Exp. 3 and 4)

**Fig 2:**
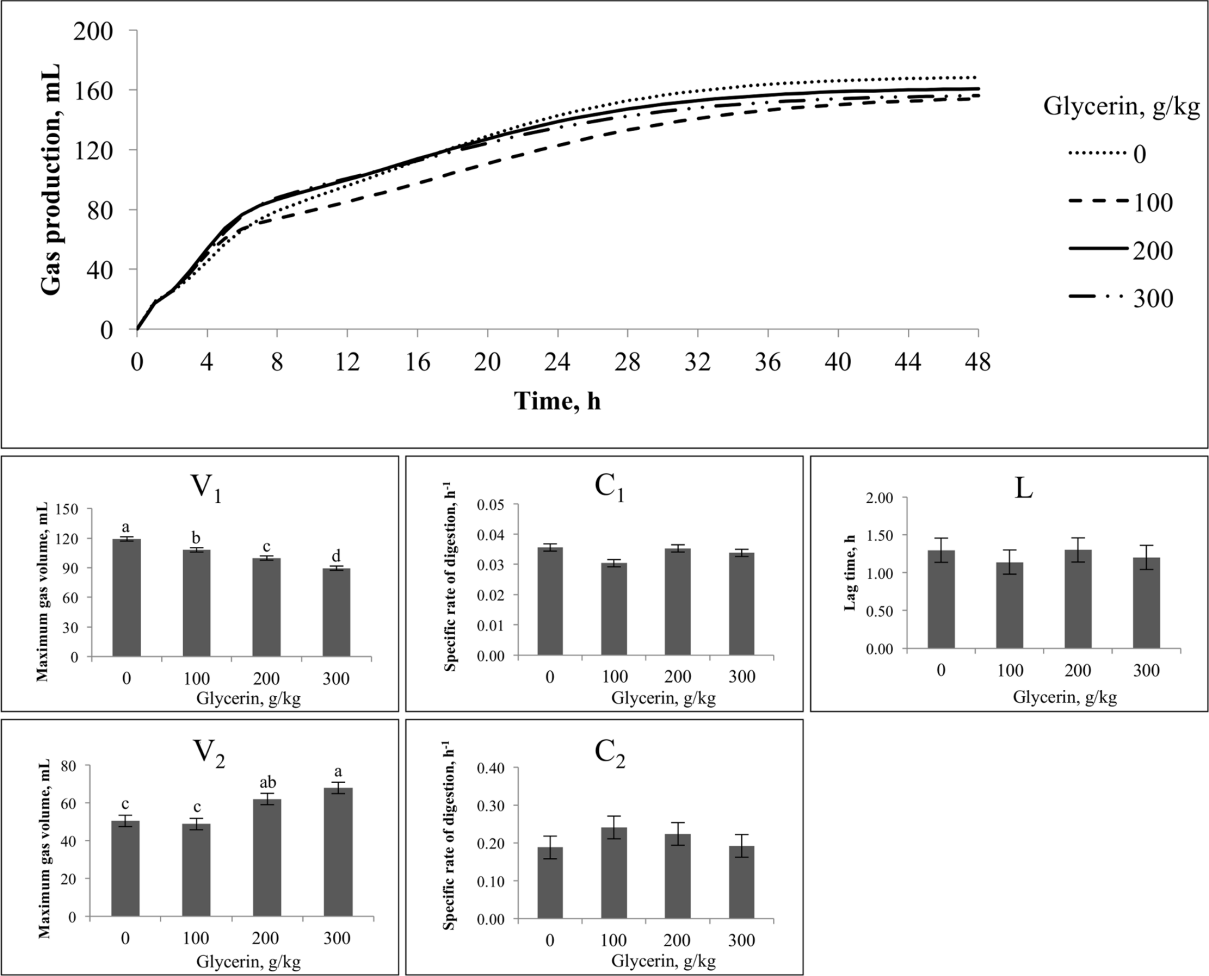
Effects of glycerin inclusion on in vitro gas production and kinetic variables^1^ of gas production in Exp. 3. ^a,b,c,d^Means with different superscripts are different (P < 0.05). ^1^V1 and V2 = Maximum gas volume of each pool, mL; C1 and C2 = Specific rate of digestion of each pool, h-1; Lt = lag time, h.

For **Exp. 3**, glycerin levels did not affect total GP, both at 24 h and 48 h incubations (*P* > 0.05; Table 4). Lag time and specific rates of digestion for both pools also did not differ among treatments (*P* > 0.05; Fig 2). The control treatment had the greatest GP for the first pool, decreasing as glycerin proportion increased (*P* < 0.05). The second pool however, had increased GP as the proportion of glycerin increased, especially when levels went from 100 to 300 g/kg of glycerin inclusion (*P* < 0.05). For **Exp. 4**, glycerin inclusion did not affect productions of CH_4_ in mL/L and mL/g of DM, and CO_2_ in mL/L and mL/g of DM (*P* > 0.05), which averaged 6.01 ± 0.97 mL/L, 9.65 ± 3.73 mL/g DM, 30.0 ± 3.31 mL/L, 48.0 ± 9.64 mL/g DM, respectively.

**Table 4:** Effects of dietary glycerin inclusion on total gas production (GP), and enteric methane (CH_4_) and carbon dioxide (CO_2_) production in GP systems.

Item^1^	Glycerin, g/kg				SEM	*P*-value	
	0	100	200	300		Linear	Quadratic
**Exp. 3**							
24h total GP, mL/g DM	280	262	274	266	29.9	0.82	0.88
48h total GP, mL/g DM	340	312	324	311	37.5	0.67	0.84
**Exp. 4**							
CH_4_, mL/L	55.2	59.1	60.3	66.1	4.44	0.11	0.83
CH_4_, mL/g DM	8.39	10.6	8.37	10.9	1.49	0.44	0.92
CO_2_, mL/L	299	308	296	293	12.5	0.60	0.63
CO_2_, mL/g DM	45.1	55.3	40.9	47.5	4.93	0.75	0.71

^1^DM, dry matter; SEM, standard error of the mean.

The effects of glycerin inclusion in beef cattle finishing diets on ruminal variables are presented in Table 5. For **Exp. 3,** glycerin levels did not affect pH, NH_3_-N, and total VFA (*P* > 0.05), which averaged 5.85 ± 0.21, 28.8 ± 4.33 mL/g DM, and 6.64 ± 1.84 mM/g DM, respectively. The inclusion of glycerin linearly decreased acetate concentration (*P* = 0.03) and acetate: propionate ratio (*P* = 0.04). Additionally, increasing levels of glycerin increased valerate concentration (*P* < 0.01). Also, glycerin inclusion did not affect propionate, butyrate, *iso*-butyrate, and *iso*-valerate (*P* > 0.05), which averaged 27.1 ± 2.09, 23.3 ± 2.41, 2.98 ± 0.36, and 7.27 ± 0.68 mol/100mol, respectively. For **Exp. 4**, glycerin inclusion also did not affect final pH, and total VFA (*P* > 0.05), which averaged 5.33 ± 0.13, and 20.5 ± 9.06 mL/g DM respectively. A linear decrease in acetate concentration (*P* = 0.04) was observed due to inclusion of glycerin in the diets. The inclusion of glycerin linearly increased valerate concentration (*P* < 0.01). Glycerin levels did not affect the concentrations of propionate, butyrate, *iso*-butyrate, *iso*-valerate, as well as acetate: propionate ratio (*P* > 0.05), which averaged 27.4 ± 3.95 mol/100 mol, 29.0 ± 2.80 mol/100 mol, 2.24 ± 0.34 mol/100 mol, 5.37 ± 0.87 mol/100 mol, and 1.13 ± 0.29, respectively.

**Table 5:** Effects of dietary glycerin inclusion on ruminal variables in gas production systems.

Item^1^	Glycerin, g/kg				SEM	*P*-value	
	0	100	200	300		Linear	Quadratic
**Exp. 3**							
Final pH	5.84	5.81	5.86	5.88	0.12	0.74	0.92
Total VFA, mM/g DM	6.66	7.50	6.62	6.42	0.82	0.61	0.58
VFA profile, mol/100 mol							
Acetate	34.3	33.4	32.7	31.7	0.79	0.03	0.89
Propionate	25.9	26.4	27.1	28	1.01	0.13	0.99
Butyrate	23.3	23.7	23.4	23.6	1.24	0.93	0.96
Valerate	5.94	6.19	6.48	6.79	0.12	< 0.01	0.91
*Iso-*butyrate	3.12	2.90	2.94	2.88	0.17	0.30	0.83
*Iso-*valerate	7.59	7.41	7.18	7.09	0.33	0.40	0.95
Acetate: propionate	1.33	1.26	1.21	1.14	0.06	0.04	0.99
NH_3_-N, mg/100 mL	29.4	29.2	27.7	28.7	1.79	0.65	0.75
**Exp. 4**							
Final pH	5.31	5.31	5.33	5.38	0.07	0.49	0.79
Total VFA mM/g DM	20.8	18.2	19.4	22.4	2.85	0.64	0.34
VFA profile, mol/100 mol							
Acetate	32.4	30.1	29.2	27.9	1.40	0.04	0.73
Propionate	26.9	27.1	27.0	28.1	2.12	0.71	0.84
Butyrate	27.5	29.2	30.1	30.0	1.28	0.17	0.52
Valerate	5.50	6.00	6.24	6.59	0.25	0.01	0.77
*Iso-*Butyrate	2.33	2.26	2.17	2.14	0.19	0.44	0.91
*Iso-*Valerate	5.41	5.34	5.37	5.25	0.46	0.83	0.96
Acetate: propionate	1.26	1.15	1.11	1.02	0.15	0.31	0.96

^1^DM, dry matter; NH_3_-N, ammonia nitrogen; SEM, standard error of the mean; VFA, volatile fatty acids.

### In vitro dry matter digestibility (Exp. 5)

**Fig 3:**
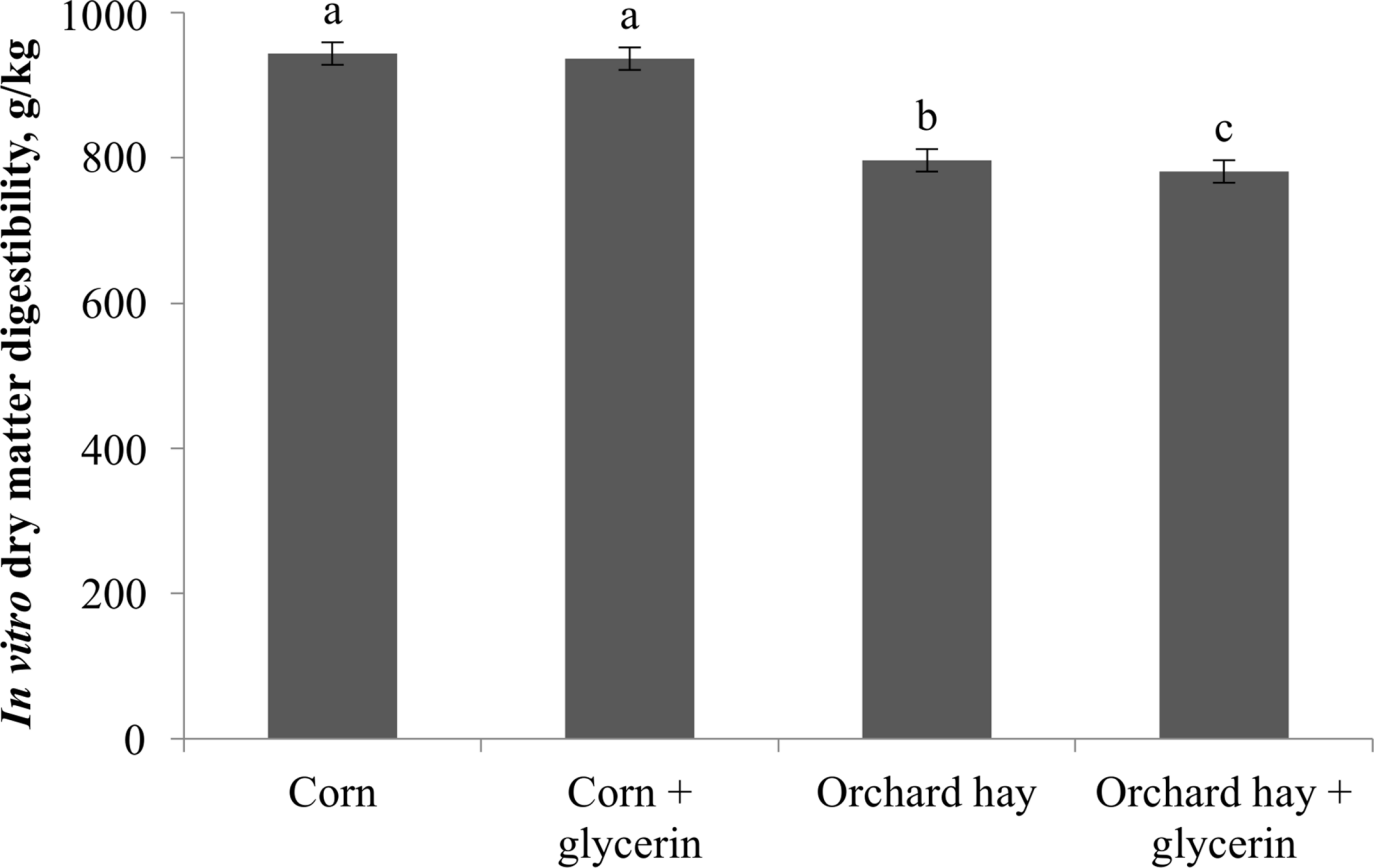
The IVDMD of individual ingredients (corn and orchard hay) and co-incubation with glycerin in Exp. 5, using two systems of four 4-L digestion vessels (Daisy^II^ system, Ankom technology, NY, USA) in four consecutive 48-h fermentation batches. ^a,b,c^Means with different superscripts are different (P < 0.05).

The inclusion of glycerin with orchard hay decreased IVDMD (*P* < 0.01; Fig 3); however, it did not change IVDMD when glycerin was added to corn-based diet.

## Discussion

### Ingredients evaluation: Corn, glycerin, and starch (Exp. 1 and 2)

The findings in this study have confirmed our hypothesis that glycerin has similar 48 h GP than starch, but lower than corn. That evidence is due to the fact that most of starch is fermented within the first 24h whereas glycerin is still fermenting after the first 24h reaching out similar total GP after 48 h fermentation. Moreover, starch had the fastest digestion rates and total gas produced until 24 h of fermentation, which might be also an explanation for the lower pH and NH_3_-N observed for starch compared to the other treatments. [5, 26]. Contradicting our hypothesis, glycerin had slower rate of GP, and greater lag time, compared to corn and starch. That may be associated to the fact that donor animals were not previously adapted to glycerin, which may have led to longer adaptation times by the rumen microorganisms, as well as longer delay in digestion observed for each pool. There is controversy regarding prior adaptation to glycerin on ruminal fermentation; Van Cleef et al. [27] reported that previous adaptation may be required to optimize glycerin utilization by ruminal microorganisms. However, previous studies observed rapid microbial adaptation in the rumen of non-adapted animals [28, 29]. Changes in VFA profile may affect ruminal GP, and fermentation to acetate yields more gas than that to propionate [30]. Thus, the greater total GP in corn treatment may be explained by the shift in VFA profile, with greater acetate concentration compared to propionate concentration. Lee et al. [5] observed that glycerin had lower total GP than corn and alfalfa using an in vitro fermentation system. Others indicated greater total in vitro GP and slower rate of GP for glycerin when compared to alfalfa, corn silage, propylene glycol, and molasses [26]. Therefore, our results indicate that despite of the fact that glycerin may have a slower rate of degradation, it has the potential to have similar fermentation pattern at 48 h.

Agreeing with our hypothesis, in vitro fermentation of glycerin had similar ME than starch. These results suggest that glycerin has similar energetic potential in the rumen than starch, probably because both are mainly fermented to propionate and have similar 48 h GP. Corn had a ME that was 7.3 and 9.7% greater than glycerin and starch, respectively, which may also suggest that glycerin has lower energy content per kg of DM than corn. The latter might be a constraint up to a certain level, especially for beef cattle finishing diets, which are fed to animals with greater energy requirements. It is worth mentioning that the equation utilized to estimate the ME takes into account the CP content of the ingredient evaluated as well as GP. Thus, the greater ME observed in corn may be related to its CP content; whereas, the other two ingredients tested (glycerin and starch) do not have CP. Furthermore, the ME observed in this study was calculated considering that 1000 g/kg of glycerin was fermented in the rumen. Rémond et al. [28] observed that glycerin may escape rumen fermentation, being absorbed by the rumen wall (up to 430 g/kg) or small intestine (up to 130 g/kg), which would yield more energy to the animal [31]. Glycerin that escapes ruminal fermentation reaches the intestine with an energy content of 18.0 MJ/kg, which is 32.4% greater than corn energy content, estimated at 13.6 MJ/kg [32]. Mach et al. [31] calculated glycerin metabolic energy (16.9 MJ/kg), considering that 500 g/kg of glycerin is fermented to propionate [1.54 MJ of ME per mol [33] in the rumen, and the other 500 g/kg escapes ruminal fermentation, being absorbed by the intestine. Working with cannulated crossbreed steers, Monnerat et al. [34] also observed greater energy levels for glycerin (15.2 MJ/kg) compared to corn, suggesting that glycerin may contribute with more energy per unit of DM than corn.

**Fig 4:**
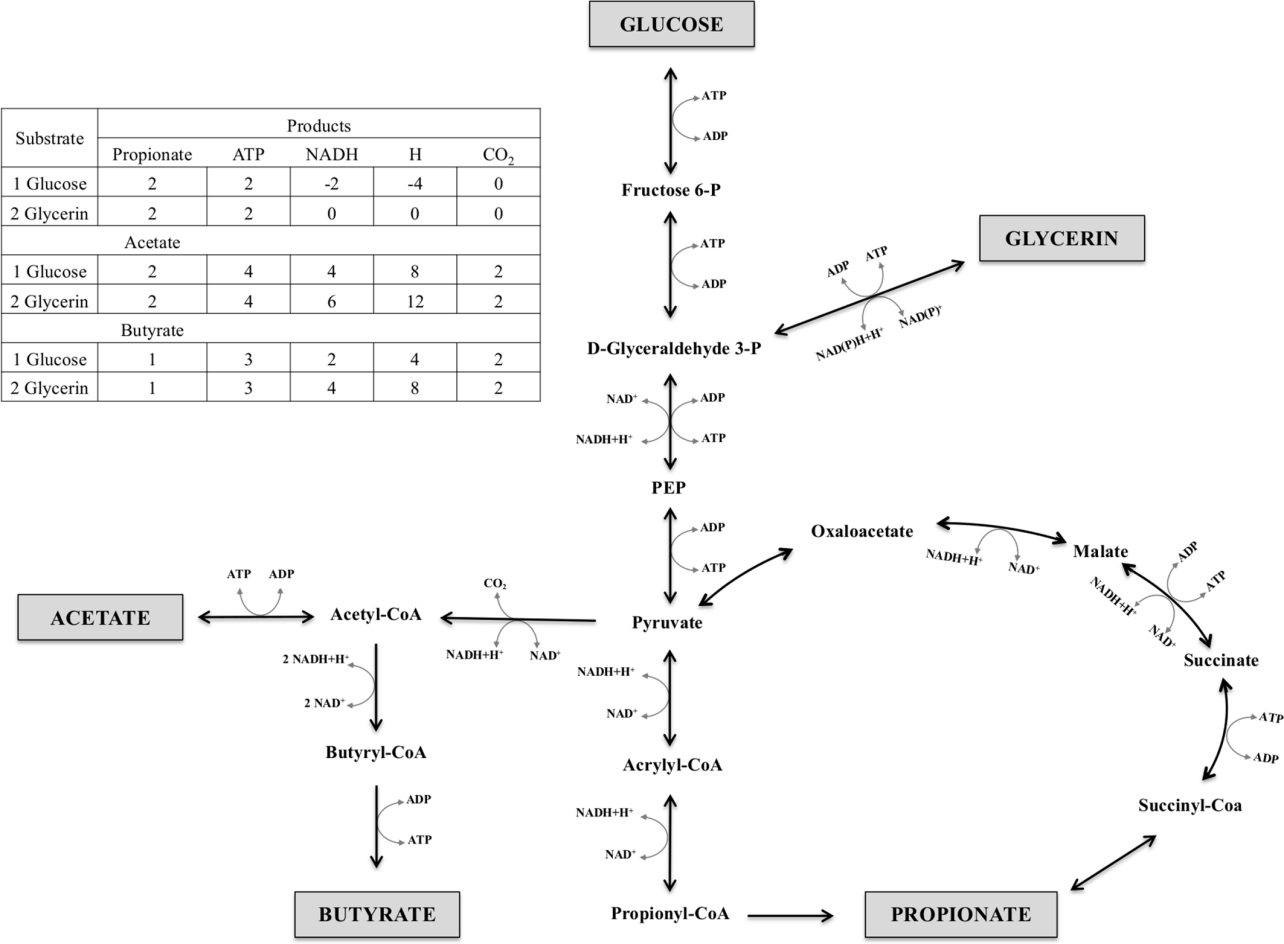
Main products of glucose and glycerin pathways to acetate, butyrate, and propionate formation^1^. ^1^Adapted from Nelson and Cox [4].

We hypothesized that glycerin would reduce the enteric CH_4_ and CO_2_ production because its fermentation leads to more propionate production than starch [3]. Pathways to propionate formation from hexoses act as a hydrogen sink [6, 7], reducing the availability of hydrogen for CH_4_ formation by changing the overall electron balance in the rumen. Furthermore, there is no CO_2_ formation in pathways to propionate production (Fig 4). As expected, glycerin treatment reduced CO_2_ (mL/L) production compared to corn and starch. However, the greater CH_4_ production for glycerin, compared to the other treatments, contradicts our hypothesis. An explanation for this result is the fact that propionate formation from glycerin does not act as a hydrogen sink [11]. For a single propionate molecule formation, pathway from glycerin releases two more hydrogen ions than that from hexoses [35]. Fig 4 illustrates glycerin and glucose fermentation pathways to acetate, butyrate, and propionate, as well as the ATP, NADH, H_2_, and CO_2_ balance of each one. Each mol of glycerin enters the glycolysis pathway as D-glyceraldehyde 3-P, releasing one mol of NADH + H^+^ and consuming one ATP. Therefore, to produce VFA, glycerin releases more hydrogen than hexoses and contributes more than corn or starch to increases on CH_4_ production. The greater butyrate concentration when glycerin is fermented also may explain the greater CH_4_ production for this treatment. Butyrate formation releases more reducing equivalents than propionate, and contributes to the enhancement of methanogenesis (Fig 4).

Regarding to total VFA concentration, the lack of differences observed between treatments for **Exp. 2** are in agreement with our hypothesis that glycerin would have similar potential ruminal fermentation compared to either starch or corn being a viable substitute for these ingredients. Moreover, it is argued that the rapid fermentation of starch could increase lactic acid concentration, which also could decrease total VFA concentration having potential detrimental effects on ruminal pH [36]. In agreement, in the current study, ruminal pH was lower for starch than for corn and glycerin. The decrease on acetate: propionate ratio for glycerin, compared to corn and starch, was expected, since the former treatment had an increase in propionate concentration at the expenses of acetate concentration. Additionally, the decrease in acetate was also associated with an increase in butyrate concentration. Acetate is on the pathway of butyrate production [28] with intense interconversion between them, therefore the increase in butyrate may be associated with the decrease in acetate concentration when glycerin was fed. Moreover, ruminal butyrate might be the main fermentation end-product of *Megasphaera elsdenii*, an important glycerin-fermenting bacteria [37]. Furthermore, the use of glycerin as energy source by *Selenomonas ruminantium* might enhance methanogenesis [38], which may be related to the greater CH_4_ production for glycerin observed in the current study.

The similar results in propionate concentration between starch and glycerin are in agreement with our hypothesis that both ingredients have similar potential for gluconeogenic precursors production in the rumen. The lower propionate concentration for corn compared to glycerin and starch in **Exp. 1** is probably associated with the fermentation of its fibrous components that lead to acetate production. Nevertheless, no differences were observed in propionate concentration in **Exp. 2**. Previous studies have observed no differences in total VFA and lower acetate concentration and acetate: propionate ratio when glycerin was fed compared to corn [5] and corn silage [26].

### Diets evaluation: Different glycerin levels in beef cattle finishing diets (Exp. 3 and 4)

We hypothesized that glycerin could replace corn and be included at up to 300 g/kg in beef cattle finishing diets without compromising GP kinetics in an in vitro fermentation system. As expected, the replacement of corn with glycerin did not change the 24 h and 48 h total GP. Avila, Chaves [39] did not find differences for 48 h total GP in an in vitro experiment when glycerin (99.5% purity) was included at up to 210 g/kg (DM basis) in diets. Despite **Exp. 1** has indicated that glycerin has slower GP rate than corn and starch, the lack of effects observed for lag time and rates of digestion with glycerin inclusion on **Exp. 3** may indicate an additive relationship between glycerin and cornstarch, which may have provided similar fermentation pattern. Hales et al. [1] observed increase on starch digestibility when glycerin was included at up to 100 g/kg in diets of beef steers. The lack of differences for total VFA, NH_3_-N and final pH with glycerin inclusion may also support our hypothesis. The fact that glycerin inclusion decreased maximum gas volume on first pool but compensated on second pool for total GP, suggests, besides the issue of microbial adaptation already discussed, that glycerin may act as a physical barrier and hinder the microorganisms access to the other components of the diet, delaying their degradation. Indeed, studies have reported decreased of fiber digestibility by glycerin inclusion [12, 40]. Moreover, on **Exp. 5** of this study, glycerin inclusion decreased the orchard hay IVDMD, but had no effects when added to corn.

The lack of effects for ruminal variables may also be the reason for partial dietary replacement of corn with glycerin did not have affect CH_4_ and CO_2_ production in this study. As discussed before, glycerin pathways to VFA release more hydrogen than that from hexoses. However, there was a decrease in acetate concentration and acetate: propionate ratio with glycerin inclusion in this study, and acetate production would result in greater release of reducing equivalents than propionate production [11]. Thus, the decreasing in acetate concentration for glycerin treatments seems to be compensated by the greater hydrogen releasing when acetate is produced from glycerin, thereby causing similar CH_4_ production among treatments. Effects of glycerin on CH_4_ and CO_2_ production in ruminants have been conflicting by in vitro and in vivo experiments. According to Avila-Stagno et al. [11], the pre-adaptation of donor animals as well as the absorption through rumen and intestine walls on in vivo experiments may be an explanation for this inconsistency. These authors observed an increase in CH_4_ production in a pre-adapted semi-continuous culture system when glycerin (995 mL/L purity) was included at up to 150 g/kg (DM basis) in forage-based diets. However, the in vitro productions of CH_4_ and CO_2_ were unaffected by glycerin inclusion using ruminal inoculum from adapted and non-adapted donor animals [27]. Similar to this study, Avila et al. [39] observed that CH_4_ production was not affected when glycerin was added at up to 210 g/kg of DM in an in vitro system. Avila-Stagno et al. [41] also noted a lack of effects in CH_4_ production when lambs were fed diets containing up to 210 g/kg (DM basis) of glycerin. Others have observed a reduction in CH_4_ and CO_2_ productions in Nelore steers when crude glycerin was included up to 300 g/kg (DM basis) in the diet [9]. As discussed before, part of the glycerin may escape rumen fermentation, being absorbed by rumen and intestine walls and consequently decreasing ruminal CH_4_ production. Nevertheless, the lack of differences in VFA concentration, total GP, and CH_4_ and CO_2_ production in this study indicates similar energy efficiency when glycerin replaces corn in beef cattle finishing diets.

### In vitro dry matter digestibility (Exp. 5)

Results from this experiment confirm our hypothesis that glycerin addition would not impact the corn IVDMD. However, adding glycerin reduced the IVDMD of orchard hay. These results suggest suppression in fiber digestibility when glycerin is added in forage feedstuffs. In the study by Roger et al. [13] an inhibition in growth and activity of cellulolytic bacteria and anaerobic fungal species were observed when glycerin was added at a concentration of 50 g/kg DM. Therefore, the inhibition of cellulolytic activity could affect fiber digestion, and consequently decrease fiber digestibility in forage based diets [12]. However, previous studies have had lack of effects in ruminal DM and NDF digestibility on in vitro studies [3, 42], or even a quadratic increase in apparent DM and NDF digestibility when glycerin was included in high concentrate diets for finishing steers [3]. According to these authors, glycerin fermentation characteristics as microbial adaptation, fast ruminal turnover [2], glycerin additive relationship with starch digestion [29, 43], as well as high VFA production [1] may stimulates DM digestion.

## Conclusion

Compared to starch, glycerin had slower rate of degradation but similar 48 h total gas production. Furthermore, glycerin and starch had similar metabolizable energy, which suggests that glycerin may be used as alternative energy source in beef cattle finishing diets.

Despite lower dry matter digestibility when glycerin was added to orchard hay-based diet, no negative effects on ruminal fermentation, total gas production, and enteric CH_4_ production were observed when glycerin was included in beef cattle finishing diets. Therefore, our results suggest that glycerin has similar energy efficiency when used in replacement of corn and included at up to 300 g/kg in beef cattle finishing diets.

## Supporting information

S1 Table Raw data. (XLSX)

## Abbreviations

aNDFom: neutral detergent fiber assayed with a heat stable amylase and expressed exclusive of residual ash
CH_4_: methane
CO_2_: carbon dioxide
CP: crude protein
DM: dry matter
EE: ether extract
GP: gas production
IVDMD: in vitro dry matter digestibility
ME: metabolizable energy
N_2_: nitrogen
NH_3_-N: ammonia-nitrogen
OM: organic matter
VFA: volatile fatty acids
